# Alexander Disease Mutations Produce Cells with Coexpression of Glial Fibrillary Acidic Protein and NG2 in Neurosphere Cultures and Inhibit Differentiation into Mature Oligodendrocytes

**DOI:** 10.3389/fneur.2017.00255

**Published:** 2017-06-06

**Authors:** Ulises Gómez-Pinedo, Maria Salomé Sirerol-Piquer, María Durán-Moreno, José Manuel García-Verdugo, Jorge Matias-Guiu

**Affiliations:** ^1^Neurobiology Laboratory, Neuroscience Institute, IdISSC, Hospital Clínico San Carlos, Universidad Complutense de Madrid, Madrid, Spain; ^2^Laboratory of Comparative Neurobiology, Instituto Cavanilles de Biodiversidad y Biologia Evolutiva, Universidad de Valencia, Valencia, Spain

**Keywords:** Alexander disease, glial fibrillary acidic protein, NG2, neurospheres, oligodendrocyte precursors, cathepsin, caspase-3, HSP27

## Abstract

**Background:**

Alexander disease (AxD) is a rare disease caused by mutations in the gene encoding glial fibrillary acidic protein (GFAP). The disease is characterized by presence of GFAP aggregates in the cytoplasm of astrocytes and loss of myelin.

**Objectives:**

Determine the effect of AxD-related mutations on adult neurogenesis.

**Methods:**

We transfected different types of mutant GFAP into neurospheres using the nucleofection technique.

**Results:**

We find that mutations may cause coexpression of GFAP and NG2 in neurosphere cultures, which would inhibit the differentiation of precursors into oligodendrocytes and thus explain the myelin loss occurring in the disease. Transfection produces cells that differentiate into new cells marked simultaneously by GFAP and NG2 and whose percentage increased over days of differentiation. Increased expression of GFAP is due to a protein with an anomalous structure that forms aggregates throughout the cytoplasm of new cells. These cells display down-expression of vimentin and nestin. Up-expression of cathepsin D and caspase-3 in the first days of differentiation suggest that apoptosis as a lysosomal response may be at work. HSP27, a protein found in Rosenthal bodies, is expressed less at the beginning of the process although its presence increases in later stages.

**Conclusion:**

Our findings seem to suggest that the mechanism of development of AxD may not be due to a function gain due to increase of GFAP, but to failure in the differentiation process may occur at the stage in which precursor cells transform into oligodendrocytes, and that possibility may provide the best explanation for the clinical and radiological images described in AxD.

## Introduction

Alexander disease (AxD), first described in 1949 by Alexander (1), is a rare and fatal CNS disease of genetic origin that is caused by a heterozygous mutation in the glial fibrillary acidic protein (GFAP) gene (2–4). Its pathological characteristic is the presence of inclusion bodies named Rosenthal fibers (5) that contain aggregated GFAP and small heat shock proteins, mainly α-β-crystallin and HSP27 (6, 7); additionally, some have suggested that Rosenthal fibers could also include such other proteins as vimentin, p62, or plectin (8). Clinical presentation depends on age of onset, and the most frequent is the infantile form consisting of motor impairment, cognitive decline, bulbar signs, and seizures (9–11). Although its mechanism is unknown, studies of cell lines and animal models had been suggested that AxD could activate stress response pathways within astrocytes due to increased expression of WT or mutant GFAP (12–17) that would potentially reduce proteasomal activity in cells (18) and due to oxidative stress potentially producing an antioxidant response mediated by the transcription factor Nrf2 (19, 20) However, although these models are able to replicate the astrocytic changes occurring in the disease, including formation of Rosenthal fibers, they have failed to reproduce myelin loss (21, 22). While AxD has historically been described as a disorder of myelin formation, with loss of myelin and oligodendrocytes appearing as demyelinated areas in MRI studies (23), the full mechanism underlying AxD is not yet well understood (24, 25). For that, other authors had proposed other possibilities (26). Thus, Olabarria et al. (27) have suggested that inflammatory mechanism may mediate in AxD, and Kanski et al. have shown that histone acetylation in astrocytes is an important regulator of transcription as well as alternative splicing of GFAP and have hypothesized that it could be a mechanism that could explain the disease (28).

In recent years, neurogenesis in adult mammals, including humans, has been described in the subgranular zone of the hippocampal dentate gyrus and in the subventricular zone (SVZ) (29–31). During development, oligodendrocyte precursor cells are generated in the ventricular area and subsequently migrate to the surrounding parenchyma while proliferating and acquiring such oligodendrocyte markers as NG2 and O4. In adults, oligodendrocytes are generated by different progenitors depending on the site (32–34); these progenitors include non-differentiated cells such as those present in the SVZ (35–37), especially in the context of demyelinating disorders (38). We postulate that AxD may affect adult differentiation since children are initially healthy and the disease appears later, with mutations acting upon the stage in which cells express NG2 prior to differentiation (39, 40), at this later stage, cells express mRNA-GFAP (41), but they do not normally produce GFAP, since it would have been expressed before the onset of the differentiation process.

Since pathology studies in AxD show neuronal, astrocytic, and myelinic changes, we have considered the possibility that GFAP mutations could act upon progenitor cells. To explore this possibility, we have analyzed the changes resulting from transfection of mutant GFAP during the neurosphere differentiation process.

## Materials and Methods

### Animals

Two-month-old CD1 Swiss male mice obtained from Charles Rives Laboratory (Barcelona, Spain) were used in this study (*n* = 12; weight = 30 g). All experiments were carried out in accordance with guidelines for animal experimentation under Spanish law (RD 1201/2005) and European directives (86/609/EEC).

### Plasmids: Procurement, Site-Directed Mutagenesis, and Purification

We selected a representative set of hGFAP mutations to study their effects, choosing high-incidence mutations affecting different protein domains. pcDNA 3.1 plasmid (Invitrogen) was used for eukaryotic expression assays. It contains the CMV promoter, which confers ubiquitous expression. Neurospheres were transfected with the pcDNA3.1 empty vector (the transfection control), hGFAP_WT (the wild type), hGFAPR88C, and constructs provided by Dr. Michael Brenner (NINDS, NIH, MD, USA): hGFAPR79H, hGFAPR239H and hGFAPR416W. Site-directed mutagenesis was performed to generate the hGFAPR88C construct. Complementary primers (see below) containing the C262T mutation were used for PCR amplification of the pcDNA3.1 plasmid. For this process, we used Pfu Turbopolymerase (Stratagene) according to the manufacturer’s instructions.

hGFAPC262TF: 5′CATCGAGAAGGTTTGCTTCCTGGAACA 3′.
hGFAPC262TR: 5′CTGTTCCAGGAAGCAAACCTTCTCGATG 3′.

Constructs were amplified in *E. coli* and subsequently tested by analyzing their restriction patterns and using DNA sequencing. Afterward, each construct was purified using the Midiprep^®^ system (Qiagen).

### Adult SVZ Neurosphere Primary Culture

Neural stem cells were isolated from the microdissected SVZs of two-month-old CD1 male mice. Animals were killed by cervical dislocation, and their brains promptly removed. SVZs were dissected as previously described by Morshead et al. (42) and incubated in a 0.9 mg/ml papain solution (Worthington Ref. LS-003119) for 40 min at 37°C. Papain solution was then removed by centrifugation and inactivated by adding a control medium, consisting of DMEM/F12 (Gibco) supplemented with glucose (Panreac; Ref. 141341-1210), NaHCO_3_ (Gibco; Ref. 25030-024), 1 M HEPES (Gibco; Ref. 15630-049), l-glutamine (Gibco; Ref. 25030-024), antibiotic–antimycotic (Gibco; Ref. 15240-062), and hormone mix [apo-Transferrin (Sigma; Ref. T-2252), insulin (Sigma; Ref. I-2767), putrescine (Sigma; Ref. P-7505), progesterone (Sigma; Ref. P-8783), and sodium selenite (Sigma; Ref. S-9133)]. SVZs were mechanically disaggregated and filtered through a 70 µm cell strainer. Cells were plated and cultured in complete medium [control medium supplemented with 10 ng/ml of FGFb (Sigma; Ref. F0291) and 20 ng/ml of murine EGF (Gibco; Ref. 53003-018)]. The culture was incubated at 37°C in a 5% CO_2_ atmosphere.

Primary neurospheres (passage 0; P0) forming in the first week of cell culture were collected, enzymatically dissociated and replated onto uncoated 6-well dishes at a density of 10,000 cells/cm^2^. Following this method, neurospheres were subsequently passaged every 7 days.

### Transfection

Neurospheres were transfected 5 days *in vitro* after passage 6–7 by means of nucleofection technology (Amaxa Nucleofector II, Lonza), using program A-33 and following the manufacturer’s instructions. Each 75 cm^2^ flask was transfected using 4 µg of plasmic DNA and cultured with complete medium. To estimate transfection efficiency, we used the pMAX-GFP plasmid supplied in the Mouse Neural Stem Cell Amaxa Nucleofector^®^ kit (Lonza).

For differentiation experiments, 36–48 h after nucleofection, neurospheres were seeded on poly-d-lysine (Sigma) coated coverslips in differentiation media, where growth factors were withdrawn and 1.5% FBS was added. Samples were analyzed at days 3 and 7 under differentiation conditions.

### Immunocytochemistry

Cells from neurosphere differentiation cultures were fixed in 4% PFA with a 30% sucrose solution for 30 min at 37°C. For immunocytochemistry, cultures were preincubated for 1 h in blocking solution (10% goat serum, 0.1% Triton X-100, BSA), followed by overnight incubation with the appropriate primary antibody at 4°C. The following primary antibodies were used: mouse anti-hGFAP (1:500, Sternberger Monoclonal), chicken anti-vimentin (1:200, Millipore), rabbit anti-NG2 (1:200, Millipore), mouse anti-Olig2 (1:200, Millipore), chicken anti-Tuj1 (1:200, Millipore), rabbit anti-active caspase-3 (1:200, Abcam), rabbit anti-HSP27 (1:200, Abcam), and rabbit anti-cathepsin (1:200, Abcam). The corresponding secondary antibodies were incubated for 2 h (Alexa-Fluor 405, 488, 555, or 647 goat anti-mouse, chicken or rabbit; 1:500; Invitrogen), followed by incubation with DAPI (1:1,000, Sigma) for 10 min and rinsing before being mounted on glass slides with Fluorsave (Calbiochem). Analyses were performed with a Nikon *80i* fluorescence microscope at 40× or 63× magnification.

### Statistical Analysis

For statistical analysis, up to four coverslips from two independent experiments were counted for each condition using the *Nikon 80i* microscope at magnifications of 40× or 63×. More than 150 transfected cells were counted per coverslip. We performed a statistical analysis to determine the percentage of each phenotype present in transfections of each plasmid. Data were analyzed using one-way analysis of variance followed by a Tukey Multiple Comparisons test. All values are presented as mean ± SE. Statistical significance was set at *p* < 0.05.

## Results

### GFAP Mutations Result in Up-Expression of GFAP

**Figure 1:**
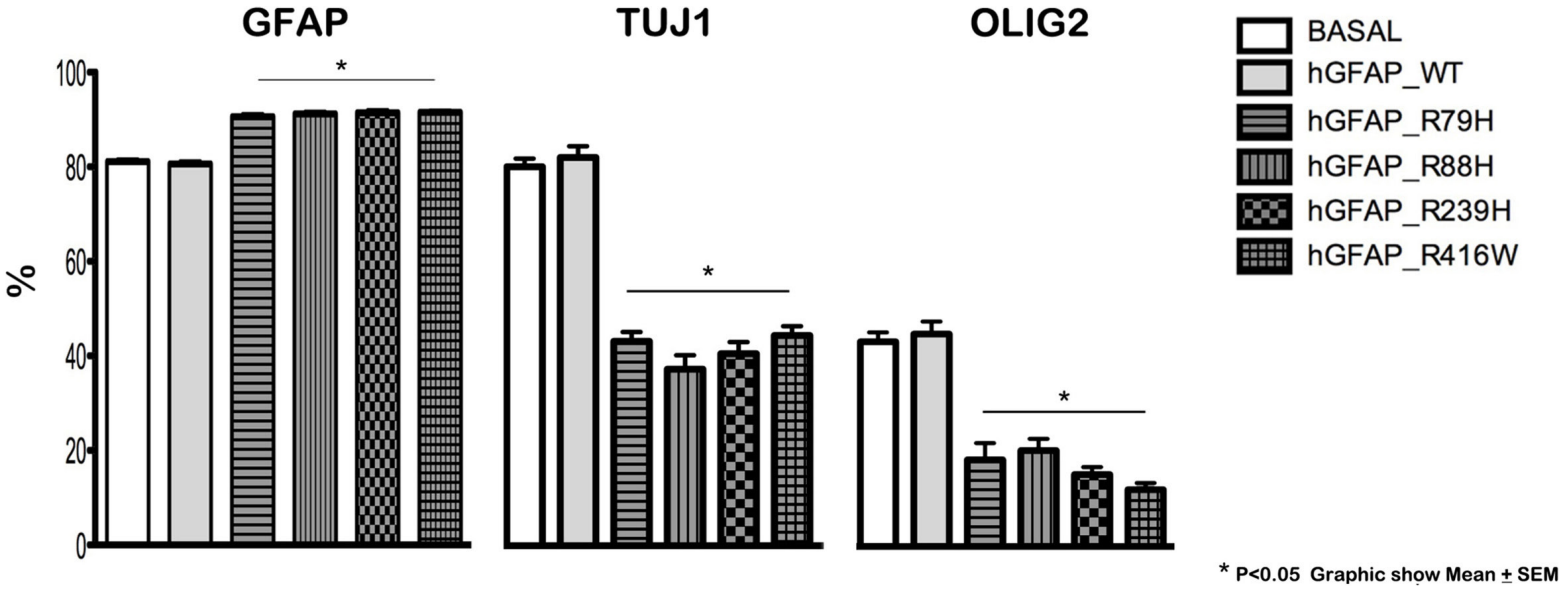
Altered cell differentiation. Changes in cell differentiation are apparent at 7 days after transfection. Percentages of cells undergoing differentiation were similar between cultures transfected with the wild-type hGFAP protein and those observed under normal conditions. Cultures containing different transfected glial fibrillary acidic protein (GFAP) mutations exhibited higher percentages of GFAP-expressing cells as well as lower percentages of differentiated oligodendrocytes (Olig2) and neurons (Tuj1). These data are statistically significant (*p < 0.05).

Transfection produced cells that differentiate into new cells marked simultaneously by GFAP and NG2 and displaying GFAP abnormalities. GFAP appeared as a protein with an anomalous structure that formed clots and aggregates and was distributed throughout the cytoplasm of the new cells; this finding was not observed in non-transfected cells (Figure S1 in Supplementary Material). Figure 1 shows a significant decrease in Olig2 and TuJ1 and a significant rise in GFAP expression in cells transfected with mutant proteins compared to the hGFAP_WT and empty plasmid groups; this suggests that mutations may interfere with neurosphere differentiation into oligodendrocytes and neurons. These changes occurred with all studied mutant forms. The reduction in oligodendrocyte and neuron markers was more prominent than the increase in GFAP, and it may therefore play a prominent role in the abnormal differentiation process caused by mutations.

### Significant Increases in Cells Expressing NG2 during Differentiation Were Found in the Mutant Protein Transfection Group

**Figure 2:**
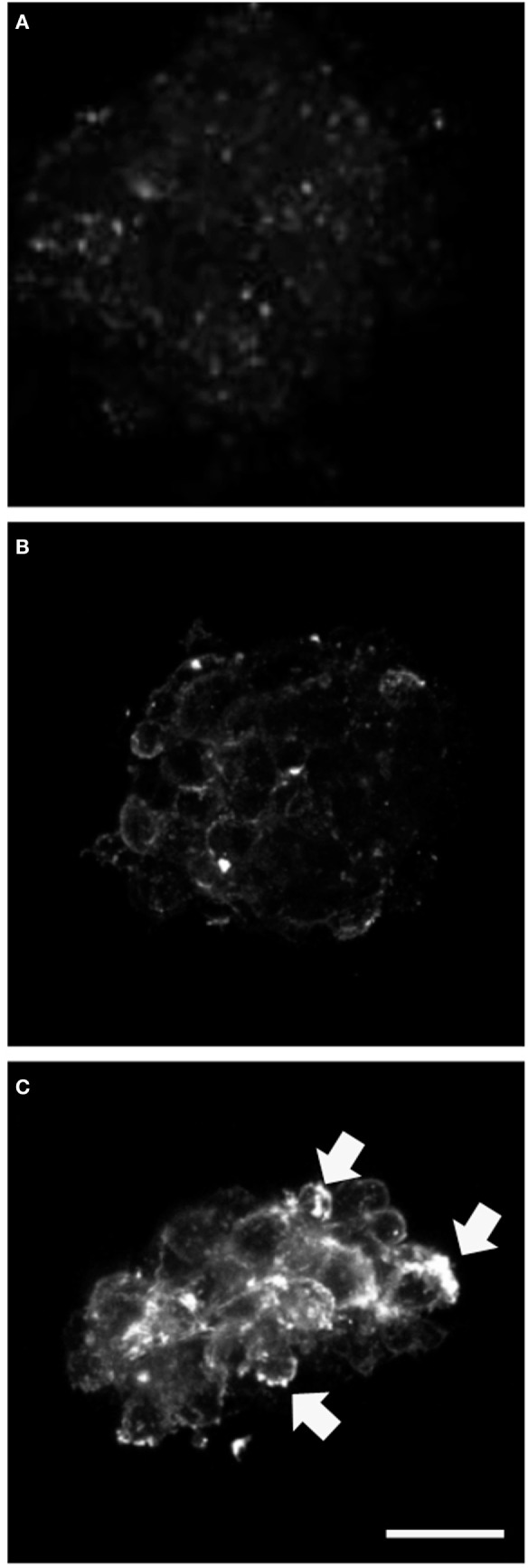
Alterations in NG2 expression. Confocal microscopy images of neurospheres undergoing differentiation (3 days). Cultures transfected with mutant glial fibrillary acidic protein (GFAP) exhibited increased expression of NG2; the protein was anomalous and formed precipitates in the cell membrane [**(C)**, arrows], compared to observations in neurospheres transfected with GFAPwt **(B)** or neurospheres under normal conditions **(A)**. Bar = 20 µm.

We analyzed expression of the progenitor cell markers vimentin, nestin, and NG2 and observed significantly higher NG2 expression at day 3 of differentiation in neurospheres transfected with mutant GFAP, compared to those transfected with hGFAP_WT or empty plasmid (Figure 2). However, no differences in expression were found for vimentin and nestin. Figure S2 in Supplementary Material shows that the NG2/Vim ratio is significantly greater for transfected AxD mutations than for transfected hGFAP_WT or empty plasmid neurospheres at day 3; this also occurred with the NG2/GFAP ratio. In contrast, the PAX3 to GFAP marker expression ratio did not differ (Figure S3 in Supplementary Material). Researchers observed high numbers of NG2+/GFAP+, but not NES+ or Vim+ cells. Percentages of GFAP+ and NG2+ cells increased with additional days of differentiation (data not shown). The expression of GFAP that appears after transfection and in the differentiation is more than four times that found in WT but is variable depending on which is the mutation transfected (date not shown). The relationship between the expression of GFAP and NG2 is also variable depending on the severity of each mutation (data not shown).

### Increased Caspase-3 Expression Was Observed during Differentiation

**Figure 3:**
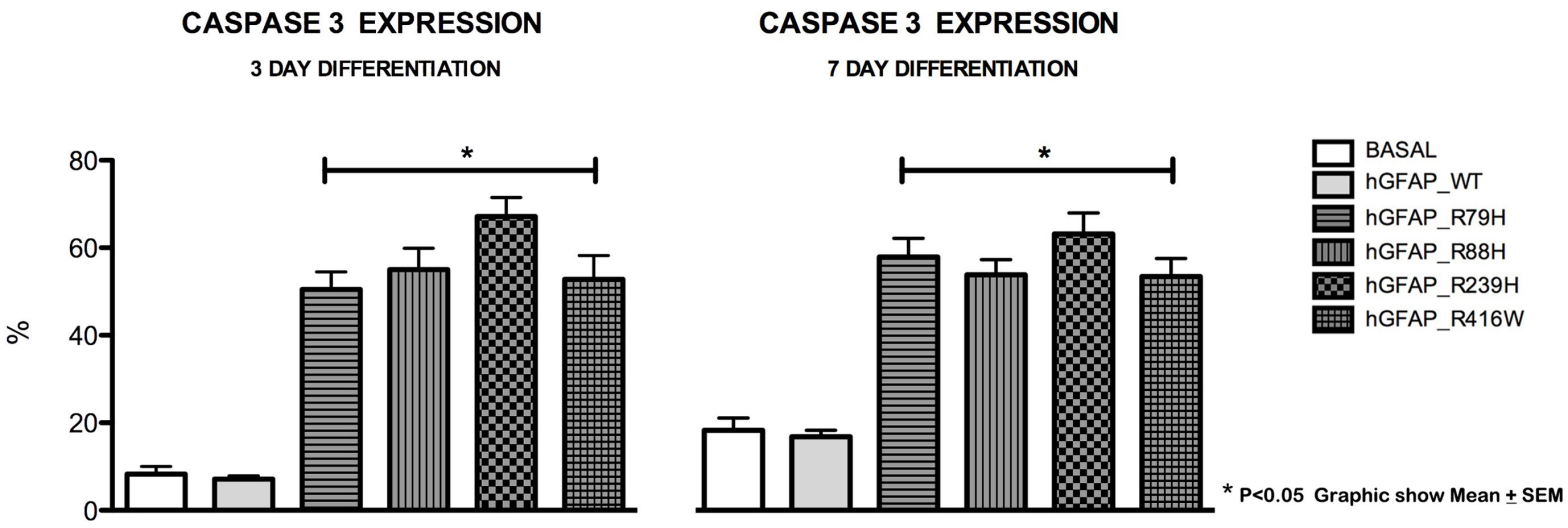
Graphs of caspase-3 expression after transfection. Cultures transfected with mutant glial fibrillary acidic protein (GFAP) protein displayed significant increases in expression of caspase-3, a marker for cell death by apoptosis, at 3 and 7 days. The level of caspase-3 was at least three times higher than those measured in the normal culture and in the culture with the WT protein. There is a direct correlation between cell death, expressed as percentage of caspase-3, and presence of mutant proteins (*p < 0.05).

**Figure 4:**
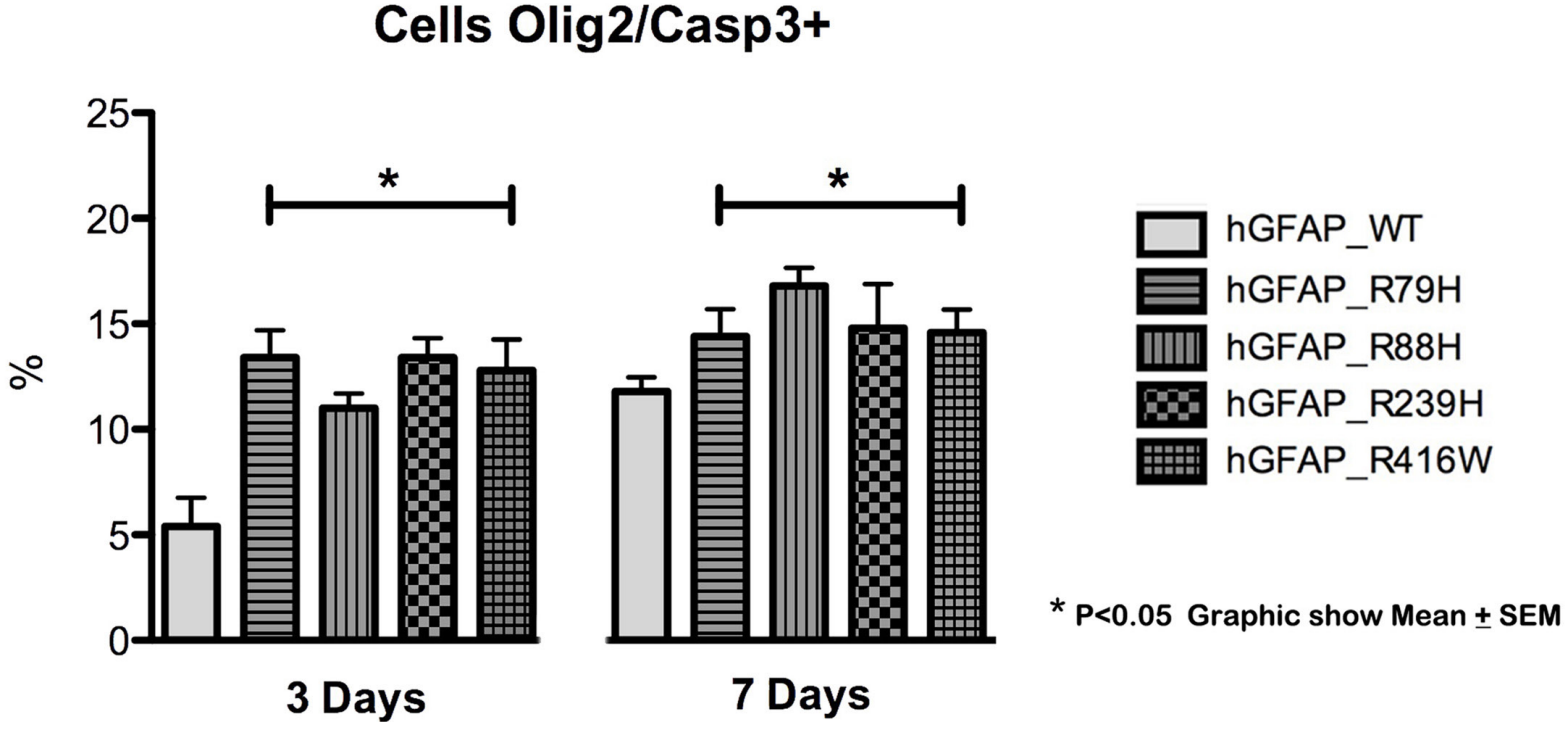
Graphs of caspase-3 expression in the oligodendroglial lineage. Increased cell death was observed in cells transfected with mutant glial fibrillary acidic protein (GFAP) at days 3 and 7. The increase in cell death was more marked on day 3 in cells expressing Olig2, an oligodendrocyte marker (*p < 0.05). On day 7, the percentage of cell death was lower, but the difference was still statistically significant (*p < 0.05). This indicates that cell death occurs more frequently in early stages of cell differentiation.

We analyzed caspase-3 expression at days 3 and 7 of differentiation and observed that transfection of the mutant protein elicited significantly augmented expression of caspase-3. This was not apparent with transfections of hGFAP_WT or empty plasmid (Figure 3). Increases in caspase-3 were significantly greater (*p* < 0.05) in VIM+ and NG2+ cells compared to those in the hGFAP_WT group, but no significant differences were observed for Pax6+ cells (Figure S4 in Supplementary Material). Caspase-3 was colocated with VIM+ and NG2+ cells and to a lesser extent with Pax6/VIM cells. Caspase-3 levels were also significantly higher on days 3 and 7 of differentiation in Olig+ cells transfected with mutant GFAP than in the hGFAP_WT group (Figure 4).

### Expression of Cathepsin D and HSP27 Rose during Differentiation

**Figure 5:**
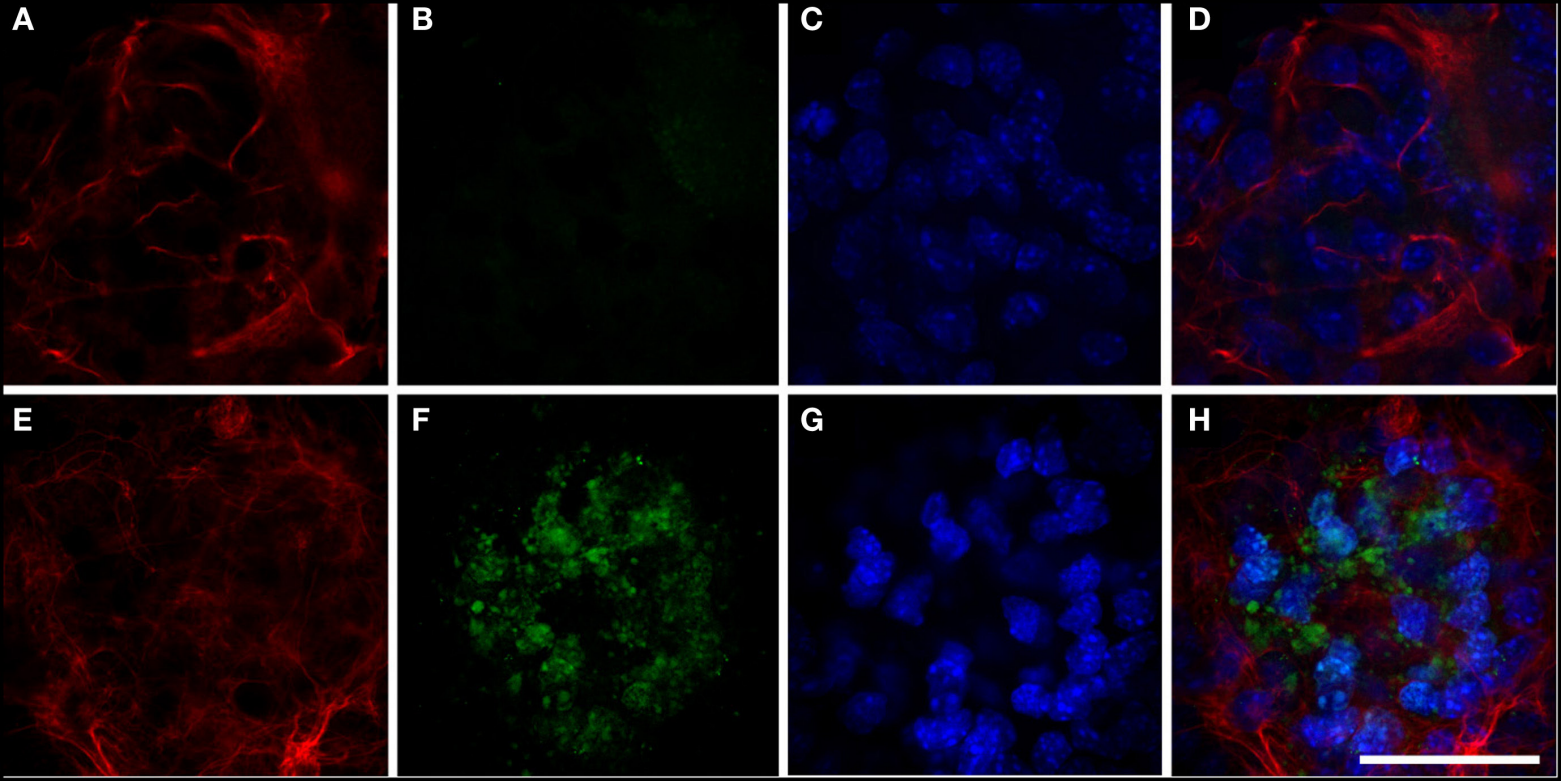
Expression of cathepsin. **(A–D)** Neurosphere transfected with hGFAP_WT protein. Expression of cathepsin is very low **(B)**; glial fibrillary acidic protein (GFAP) markers are observed in fine, well-organized cells **(A)**. **(E–H)** Neurosphere transfected with mutant hGFAP, showing expression of filamentous GFAP **(E)**. Expression of cathepsin in this case appears as markings on small vesicles resembling lysosomes distributed throughout the neurosphere. Nuclei are apparent in panels **(C**,**G)**; panels **(D**,**H)** show the sum of all channels. Bar = 50 µm.

**Figure 6:**
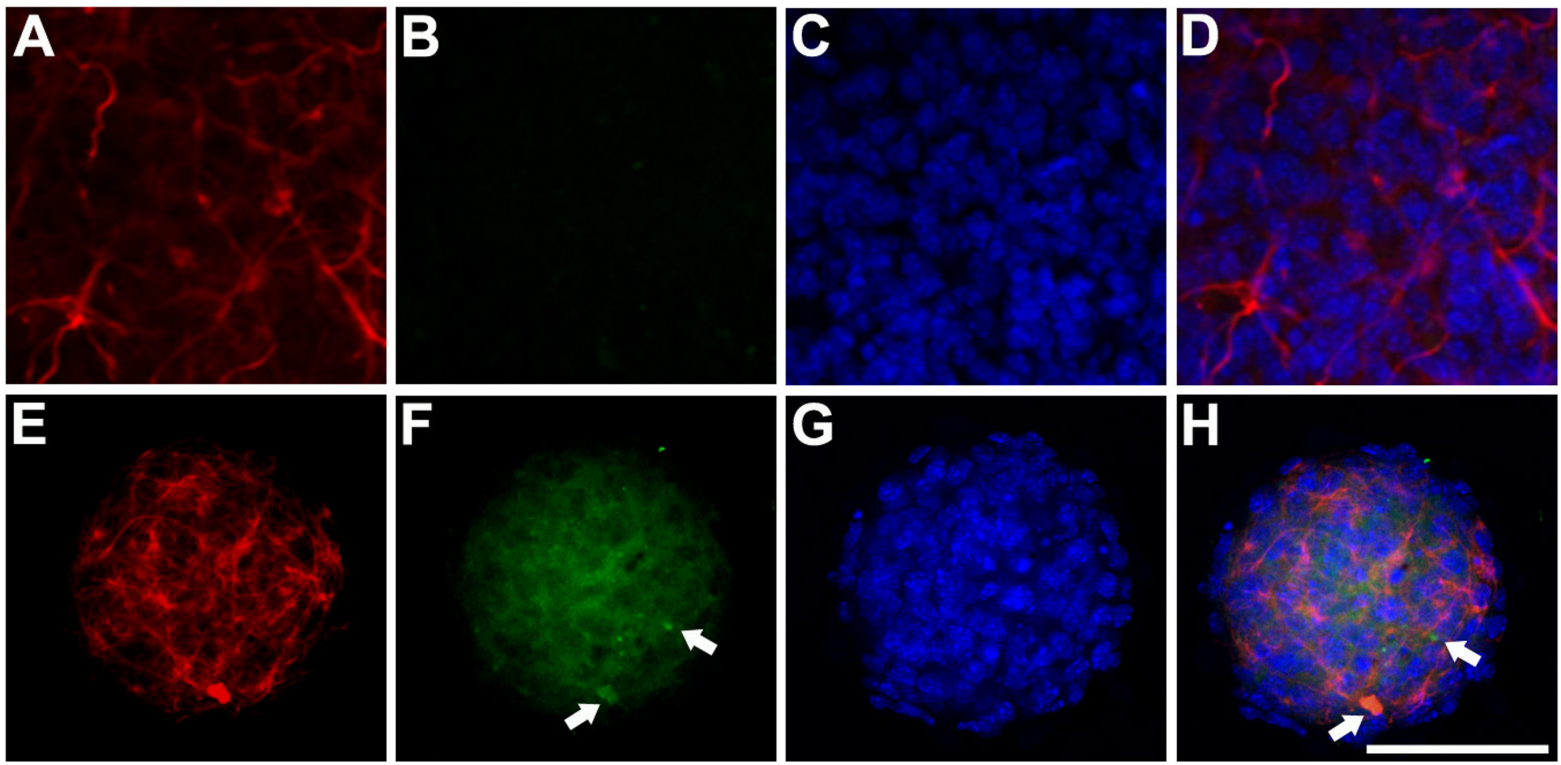
Expression of HSP27 Anti-HSP27/glial fibrillary acidic protein (GFAP) immunocytochemistry study of transfected neurospheres after 3 days of differentiation. **(A–D)** Neurosphere transfected with the hGFAP_WT protein, showing faint marking for HSP27 protein and expression of filamentous GFAP. **(E–H)** Neurosphere transfected with mutant hGFAP, revealing high expression of HSP27 protein in the form of filamentous aggregates that colocate with hGFAP expression (arrows). Bar = 50 µm.

We analyzed cathepsin D expression at day 3 of differentiation and observed that transfection of the mutant protein resulted in a significant increase in expression of this protein, which was not observed with transfection of hGFAP_WT (Figure 5). Increased cathepsin D expression indicates a lysosomal response; cathepsins have been implicated in the cell’s defense mechanisms against anomalous proteins that may contribute to cell damage (40). We also detected significantly higher levels of HSP27 expression than in cells transfected with hGFAP_WT or empty plasmid (Figure 6), suggesting that the mutation augmented the expression of small heat shock proteins, as has already been described in AxD.

## Discussion

Our study provides evidence that AxD may be able to affect myelin production since mutations act on oligodendrocyte differentiation. Our data indicate that GFAP-NG2 cells, those expressing both NG2 and GFAP, are more numerous in the mutation group than in the WT cell line.

Astrocytes play an important role in central nervous system function whether under normal or pathological conditions. Adult astrogliogenesis occurs in neurodegenerative disorders and relies on changes in GFAP expression. Increased levels of GFAP expression are associated with more severe reactive gliosis in a variety of neuropathological conditions and in gliomas (43, 44); cell pathology studies may reveal inclusion bodies—Rosenthal fibers (5)—containing ubiquitinated GFAP aggregates; these inclusion bodies have been observed in syringomyelia, multiple sclerosis (45), and certain subtypes of glioma as well as in AxD. Myelin loss is a characteristic diagnostic finding in AxD. Extensive cerebral white matter changes with frontal predominance with usual involvement of the basal ganglia, and thalamus is typical of radiological images in AxD; it is very intense in cases of great survival capacity (46). These imaging results are very suggestive of AxD and are not seen in other conditions related with Rosenthal bodies as some forms of gliomas. The past few years have advanced our understanding of the impact of GFAP levels on the disease (47), but the cause of demyelination remains unclear.

Glial fibrillary acidic protein has an anomalous structure, and it is distributed as cytoplasmic inclusions and aggregates. Failure of non-differentiated cells to transform into adult oligodendrocytes would explain loss of myelin in this disease. Incomplete differentiation into oligodendrocytes is probably what perpetuates high levels of NG2 expression; this situation arises as a means of compensating for the absence of necessary oligodendrocytes, since higher numbers of NG2 cells will differentiate into oligodendrocytes in the context of a demyelinating process than under normal conditions. If GFAP-NG2 cells are unable to produce oligodendrocytes, this could explain why their numbers rise as differentiation into other lineages decreases (38) These findings have been reported by previous studies performed with other non-differentiated cell lines (48). During differentiation, cells expressing both NG2 and GFAP proteins show diminished expression of vimentin and nestin. These data are concordant with those of Hsiao et al. (49), who observed that transfection of the mutant protein R232C in cells with increased vimentin expression does not elicit the cell-level consequences appearing in AxD because GFAP aggregates may be decreased by vimentin (50). Taking into account that GFAP-null mice are essentially normal, and other intermediate filaments, such as vimentin, can replace most GFAP functions (51), Vim+ cells may be more resistant to increased GFAP expression.

Different studies using cell and animal models (12, 14, 15, 52–54) have been designed to demonstrate the mechanism by which cell damage takes place so as to identify potential therapeutic agents (55, 56). As mutant GFAP forms aggregates, it sequesters HSP27 and α-β-crystallin proteins and becomes phosphorylated and ubiquinated, generating Rosenthal bodies and initiating cellular damage autophagy will probably provide the final pathway to cell death (57). It was postulated that impediments to GFAP degradation could create an imbalance between soluble and insoluble proteins (58) and lead to accumulations of such other proteins as α-β-crystallin and plectin (59). Upregulation of α-β-crystallin could also constitute a defense mechanism against cell damage (60). However, during the differentiation process in our study, we observed an increase in the expression of caspase-3. Another recent suggestion is that a C-terminal end of the molecules in the mutant protein could activate caspase-3 (61) that participates in the proteolysis of GFAP assembly (62, 63). This activation mechanism might be more frequent in cells with NG2+ or Olig2+ markers according to our data, which suggest that some cells would be more likely than NG2− cells to disappear after initiating the differentiation process.

Our data also show that the presence of small heat shock proteins in the cytoplasm is not an initial mechanism. HSP27 was up-expressed at all days of differentiation. These data, coinciding with those in the literature, indicate that small heat shock proteins, mainly α-β-crystallin, could protect against proteasomal alteration caused by GFAP aggregation (18, 61). We also found increased expression of cathepsin D, indicating that GFAP aggregates may produce a lysosomal response as others have suggested before (57, 64).

The alteration of the GFAP splicing and the variation of the different protein isoforms have been related to alterations of the white matter (65, 66). An patient with the disease with a mutation in the GFAP gene and a mutation in the HDAC6 gene was associated with a more severe phenotype of the disease and with reduced activity of HDAC6 (67).

In conclusion, our findings seem to suggest that difficulties in the differentiation are to be found in the process in which precursor cells transform into oligodendrocytes and would explain that all findings described in AxD can not be exclusively explained by a mechanism of gain of function by the increase of the expression of GFAP. And so, epigenetic (28), inflammatory (27), or posttranslational changes may be also associated (26).

## Ethics Statement

The present study complies with the ethical standards of the research committee at our center and the Declaration of Helsinki and its subsequent amendments.

## Author Contributions

Study design: UG-P, MS-P, JG-V, and JM-G; hGFAPR88C plasmid construction and transfections: MS-P; microscopy and molecular study: UG-P, MD-M, MS-P, and JG-V; statistical analysis: UG-P and JM-G; analysis of results and manuscript revision and approval: all the authors; figures: UG-P; manuscript draft: JM-G.

## Conflict of Interest Statement

The authors declare that the research was conducted in the absence of any commercial or financial relationships that could be construed as a potential conflict of interest.

## Supplementary Material

Figure S1 Confocal microscopy images showing expression of glial fibrillary acidic protein (GFAP) after transfection. **(A)** Cells transfected with wild-type protein (hGFAP_WT); **(B)** cells transfected with mutant form hGFAPR88C. After transfection, cells exhibit disorganization of the astrocyte cytoskeleton; GFAP protein aggregates are visible in the cytoskeleton [**(B)**, arrow]. Bar = 20 µm.

Figure S2 Coexpression of NG2/VIM in cell differentiation. At day 3 of cell differentiation, there was an increase in the expression of markers of glial progenitor cells in neurospheres transfected with mutant glial fibrillary acidic protein (GFAP). This increase in vimentin (VIM) in cells transfected with a mutation may serve to compensate for the functional alteration in the mutant protein. The increase in expression was statistically significant (**p* < 0.05).

Figure S3 Coexpression of PAX/glial fibrillary acidic protein (GFAP) in cell differentiation. At day 3, there were no changes or differences between transfected and non-transfected cultures for the PAX6 marker, which must be present for new neurons to be generated by astrocytes.

Figure S4 Cell death by apoptosis in glial and oligodendroglial progenitor cells. Based on the expression of caspase-3, cell death increased in cells transfected with mutant glial fibrillary acidic protein (GFAP), especially those positive for NG2 or VIM; these markers are closely associated with glial differentiation (astrocytes and oligodendrocytes). Analysis of the expression of neural progenitor transcription factor PAX6 in cells transfected with mutant protein revealed no differences between the transfected wild-type group and the normal group.
