# Supplementary figures and images for: Alexander Disease Mutations Produce Cells with Coexpression of Glial Fibrillary Acidic Protein and NG2 in Neurosphere Cultures and Inhibit Differentiation into Mature Oligodendrocytes
Source: Front Neurol. 2017 Jun 6;8:255. doi: 10.3389/fneur.2017.00255 (PMC5459916; doi:10.3389/fneur.2017.00255)

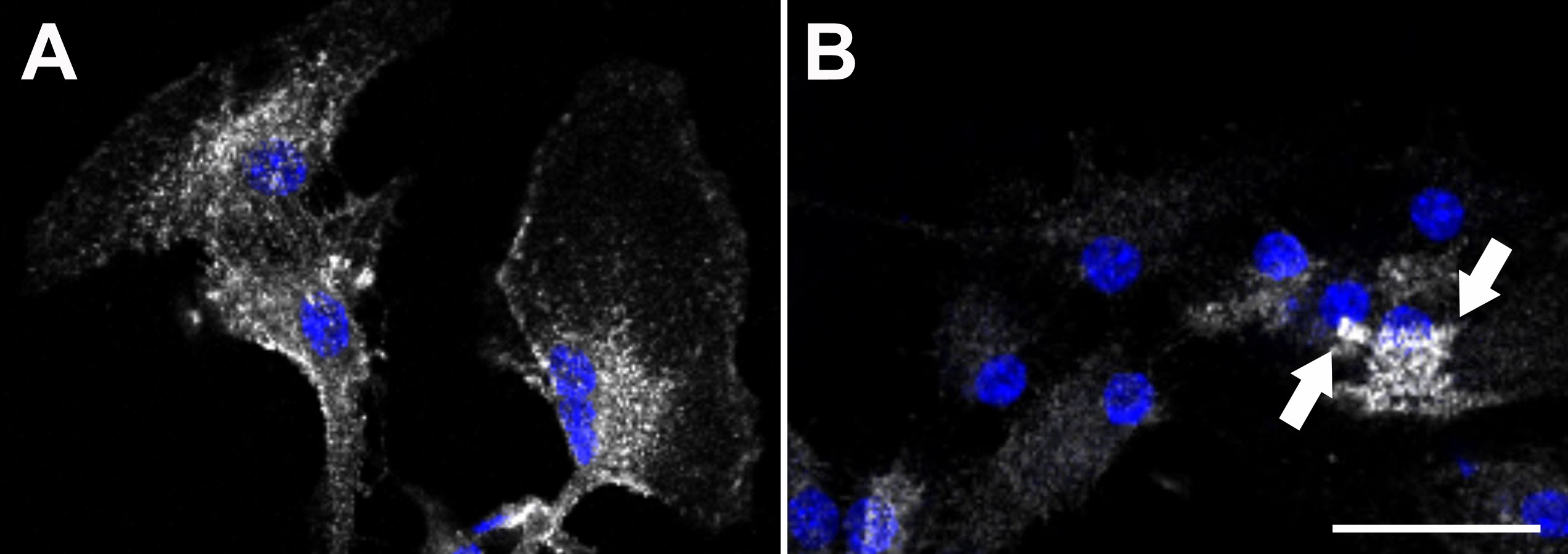

Supplement: Figure S1 — Confocal microscopy images showing expression of glial fibrillary acidic protein (GFAP) after transfection. (A) Cells transfected with wild-type protein (hGFAP_WT); (B) cells transfected with mutant form hGFAPR88C. After transfection, cells exhibit disorganization of the astrocyte cytoskeleton; GFAP protein aggregates are visible in the cytoskeleton [(B), arrow]. Bar = 20 µm. [file Image_1.JPEG]

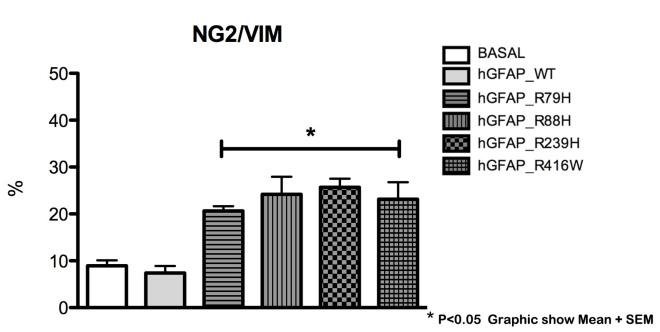

Supplement: Figure S2 — Coexpression of NG2/VIM in cell differentiation. At day 3 of cell differentiation, there was an increase in the expression of markers of glial progenitor cells in neurospheres transfected with mutant glial fibrillary acidic protein (GFAP). This increase in vimentin (VIM) in cells transfected with a mutation may serve to compensate for the functional alteration in the mutant protein. The increase in expression was statistically significant (*p < 0.05). [file Image_2.JPEG]

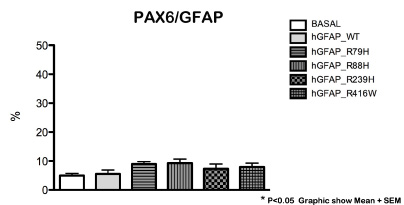

Supplement: Figure S3 — Coexpression of PAX/glial fibrillary acidic protein (GFAP) in cell differentiation. At day 3, there were no changes or differences between transfected and non-transfected cultures for the PAX6 marker, which must be present for new neurons to be generated by astrocytes. [file Image_3.JPEG]

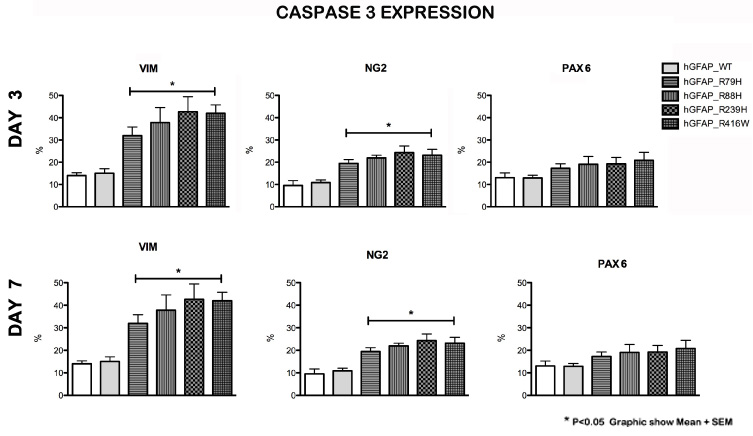

Supplement: Figure S4 — Cell death by apoptosis in glial and oligodendroglial progenitor cells. Based on the expression of caspase-3, cell death increased in cells transfected with mutant glial fibrillary acidic protein (GFAP), especially those positive for NG2 or VIM; these markers are closely associated with glial differentiation (astrocytes and oligodendrocytes). Analysis of the expression of neural progenitor transcription factor PAX6 in cells transfected with mutant protein revealed no differences between the transfected wild-type group and the normal group. [file Image_4.JPEG]
